# Single-Cell RNA-seq Reveals Angiotensin-Converting Enzyme 2 and Transmembrane Serine Protease 2 Expression in TROP2^+^ Liver Progenitor Cells: Implications in Coronavirus Disease 2019-Associated Liver Dysfunction

**DOI:** 10.3389/fmed.2021.603374

**Published:** 2021-04-22

**Authors:** Justine Jia Wen Seow, Rhea Pai, Archita Mishra, Edwin Shepherdson, Tony Kiat Hon Lim, Brian K. P. Goh, Jerry K. Y. Chan, Pierce K. H. Chow, Florent Ginhoux, Ramanuj DasGupta, Ankur Sharma

**Affiliations:** ^1^Genome Institute of Singapore, Agency for Science, Technology and Research, Singapore, Singapore; ^2^Singapore Immunology Network (SIgN), Agency for Science, Technology and Research, Singapore, Singapore; ^3^Department of Reproductive Medicine, KK Women's and Children's Hospital, Singapore, Singapore; ^4^Department of Pathology, Singapore General Hospital, Singapore, Singapore; ^5^Department of Hepato-Pancreato-Biliary and Transplant Surgery, Singapore General Hospital, Singapore, Singapore; ^6^Division of Surgical Oncology, National Cancer Centre, Singapore, Singapore; ^7^Shanghai Institute of Immunology, Shanghai JiaoTong University School of Medicine, Shanghai, China; ^8^Translational Immunology Institute, SingHealth Duke-National University of Singapore Academic Medical Centre, Singapore, Singapore; ^9^Harry Perkins Institute of Medical Research, Queen Elizabeth II Medical Centre and Centre for Medical Research, Nedlands, WA, Australia; ^10^Curtin Medical School, Curtin Health Innovation Research Institute, Curtin University, Perth, WA, Australia

**Keywords:** SARS-CoV-2, COVID-19, ACE2, tmprss2, Trop2, liver, ScRNA-seq

## Abstract

The recent coronavirus disease 2019 (COVID-19) pandemic is caused by severe acute respiratory syndrome coronavirus 2. COVID-19 was first reported in China (December 2019) and is now prevalent across the globe. Entry of severe acute respiratory syndrome coronavirus 2 into mammalian cells requires the binding of viral Spike (S) proteins to the angiotensin-converting enzyme 2 receptor. Once entered, the S protein is primed by a specialized serine protease, transmembrane serine protease 2 in the host cell. Importantly, besides the respiratory symptoms that are consistent with other common respiratory virus infections when patients become viremic, a significant number of COVID-19 patients also develop liver comorbidities. We explored whether a specific target cell-type in the mammalian liver could be implicated in disease pathophysiology other than the general deleterious response to cytokine storms. Here, we used single-cell RNA-seq to survey the human liver and identified potentially implicated liver cell-type for viral ingress. We analyzed ~300,000 single cells across five different (i.e., human fetal, healthy, cirrhotic, tumor, and adjacent normal) liver tissue types. This study reports on the co-expression of angiotensin-converting enzyme 2 and transmembrane serine protease 2 in a TROP2^+^ liver progenitor population. Importantly, we detected enrichment of this cell population in the cirrhotic liver when compared with tumor tissue. These results indicated that in COVID-19-associated liver dysfunction and cell death, a viral infection of TROP2^+^ progenitors in the liver might significantly impair liver regeneration in patients with liver cirrhosis.

## Introduction

Since December 2019, the severe acute respiratory syndrome coronavirus 2 (SARS-CoV-2) pandemic has impacted millions of lives worldwide. As of August 23, 2020, more than 23 million people are reported to be infected, with ~5% mortalities (https://coronavirus.jhu.edu/map.html). The SARS-CoV-2 is a single-stranded RNA virus belonging to the Coronaviridae family of zoonotic viruses that infect mammals and birds (1). The novel SARS-CoV-2 was first isolated from the lung airway epithelial cells of a patient with pneumonia (2). Since then, it has been reported that SARS-CoV-2 uses receptor angiotensin-converting enzyme 2 (ACE2) for entry into human cells and utilizes transmembrane serine protease 2 (TMPRSS2) for Spike (S) Protein priming (3). SARS-CoV-2 shares ~80% sequence similarity with SARS-CoV and ~50% with Middle East respiratory syndrome coronavirus, all of which cause severe respiratory symptoms (3). Moreover, in addition to respiratory disease, SARS and MERS are known to cause liver impairments (4–6).

Importantly, SARS-CoV-2 RNA was discovered in the stool sample of the first patient in the United States, indicating gastrointestinal (GI) tract infection (7). Laboratory results of the patients in this study showed an increase in the levels of alkaline phosphatase, alanine aminotransferase, aspartate aminotransferase, and lactate dehydrogenase, indicating that the hepatic function is affected. A recent study reported 14–53% cases with higher levels of alanine aminotransferase and aspartate aminotransferase in the liver of coronavirus disease 2019 (COVID-19) patients (6, 8). Moreover, these symptoms were elevated in patients admitted to intensive care units compared with those who did not require treatment in the intensive care unit (8). Recent studies have shown how elevated alkaline phosphatase or bilirubin are indicators of SARS-CoV-2-induced liver injury. A liver function pattern in infected patients with abnormal liver function has been studied by observing the levels of alkaline phosphatase or bilirubin (9, 10). A retrospective study done on 105 patients comparing severe with mild cases concluded that patients with severe cases are more likely to have an abnormal liver function (11). It remains to be investigated whether SARS-CoV-2 directly infects liver cells. In addition, concerns have been raised on the effect of SARS-CoV-2 infection on preexisting liver conditions (6, 12–14).

Since it was reported that ACE2 and TMPRSS2 are required for the entry of SARS-CoV-2 into human epithelial cells, there have been several papers that have shown the expression of these markers in different organs. In a study consisting of human, primate, and mouse samples, ACE2 and TMPRSS2 expression was observed in the lung, gut, and nasal mucosa (15). These target markers were also expressed in human and mouse ocular cells concluding that the cornea can be potentially infected by the virus (16).

Because SARS-CoV-2 binds to ACE2 and requires TMPRSS2 for activation and previous reports have shown that the liver is one of the organs that is affected by the virus, we surveyed the human liver (from tumor and adjacent normal regions of hepatocellular carcinoma patients) by single-cell RNA-seq (scRNA-seq) to identify which cell type co-express these two genes.

Here, we report that ACE2 and TMPRSS2 are co-expressed in only one subpopulation in the human liver. Based on the expression of cell type-specific markers ALB (Albumin), KRT (Keratin), and EPCAM and the unique expression pattern of TROP2 (TACSTD2) and SOX9 (SRY-box 9), we annotated this population as liver progenitors. The results of the study suggest that the SARS-CoV-2-binding receptor ACE2 is only expressed on TROP2^high^ cholangiocyte-biased progenitors, whereas TROP2^high^ and TROP2^int^ populations express serine protease TMPRSS2. These results indicate that SARS-CoV-2 infection might preferentially infect the TROP2^high^ cholangiocyte-biased progenitor pool, thereby compromising the regenerative abilities of an infected liver and/or contributing to liver pathology (17).

## Results

### Expression of Angiotensin-Converting Enzyme 2 and Transmembrane Serine Protease 2 in Human Liver Single-Cell RNA-seq Atlas

**Figure 1:**
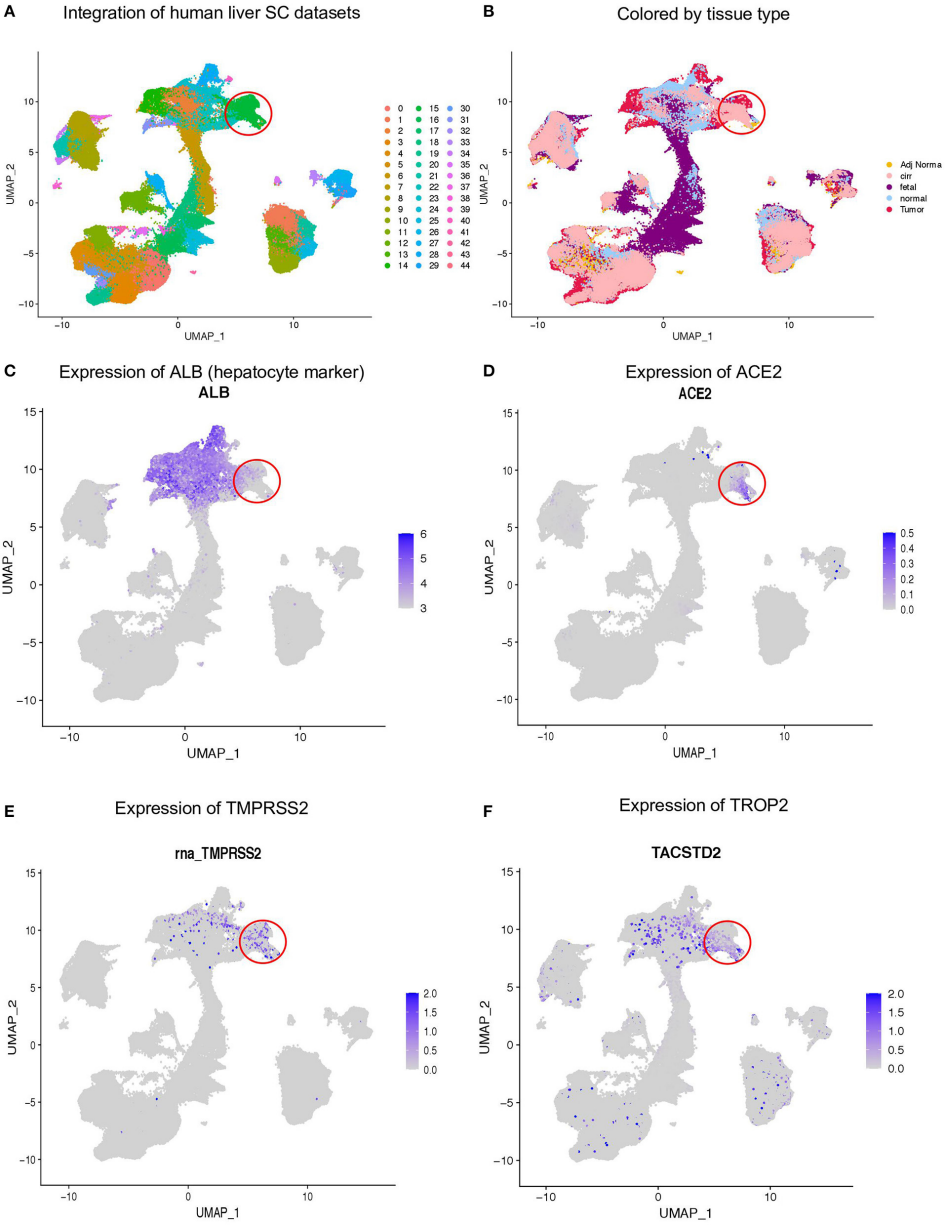
Expression of ACE2 and TMPRSS2 in human liver. **(A)** Integration of ~300,000 sc-RNA-seq libraries from fetal, adult normal, cirrhotic, and HCC patients, which identified 45 clusters in the human liver. **(B)** Louvain clusters, colored by tissue types. Expression of **(C)** ALB, **(D)** ACE2, **(E)** TMPRSS2, and **(F)** TROP2.

We performed scRNA-seq on the human liver tissue obtained from the tumors and adjacent normal tissue of hepatocellular carcinoma patients (18). In total, we analyzed ~74,000 cells and additional ~60,000 cells from the human fetal liver. Furthermore, we integrated these data with healthy (19) and cirrhotic (20) human liver scRNA-seq data. We identified ~45 clusters based on the expression of cell type-specific genes (Figure 1A). We observed integration of multiple tissue types in similar clusters indicating conservation of cell types across tissue (Figure 1B). Next, we investigated which cell types in the human liver express hepatocyte marker ALB (Figure 1C), SARS-CoV-2-binding receptor ACE2 (Figure 1D), and the priming enzyme TMPRSS2 (Figure 1E). Our analysis revealed the specific expression of ACE2 and TMPRSS2 in the ALB negative epithelial cluster. More importantly, this cluster also expresses TROP2, a gene associated with the liver epithelial progenitor population (21) (Figure 1F). This suggests that a subpopulation of liver epithelial cells expresses machinery for both SARS-CoV-2 entry (ACE2) and priming (TMPRSS2) and might be susceptible to viral infection leading to liver dysfunction.

### TROP2+ Liver Epithelial Progenitors Express Angiotensin-Converting Enzyme 2 and Transmembrane Serine Protease 2

**Figure 2:**
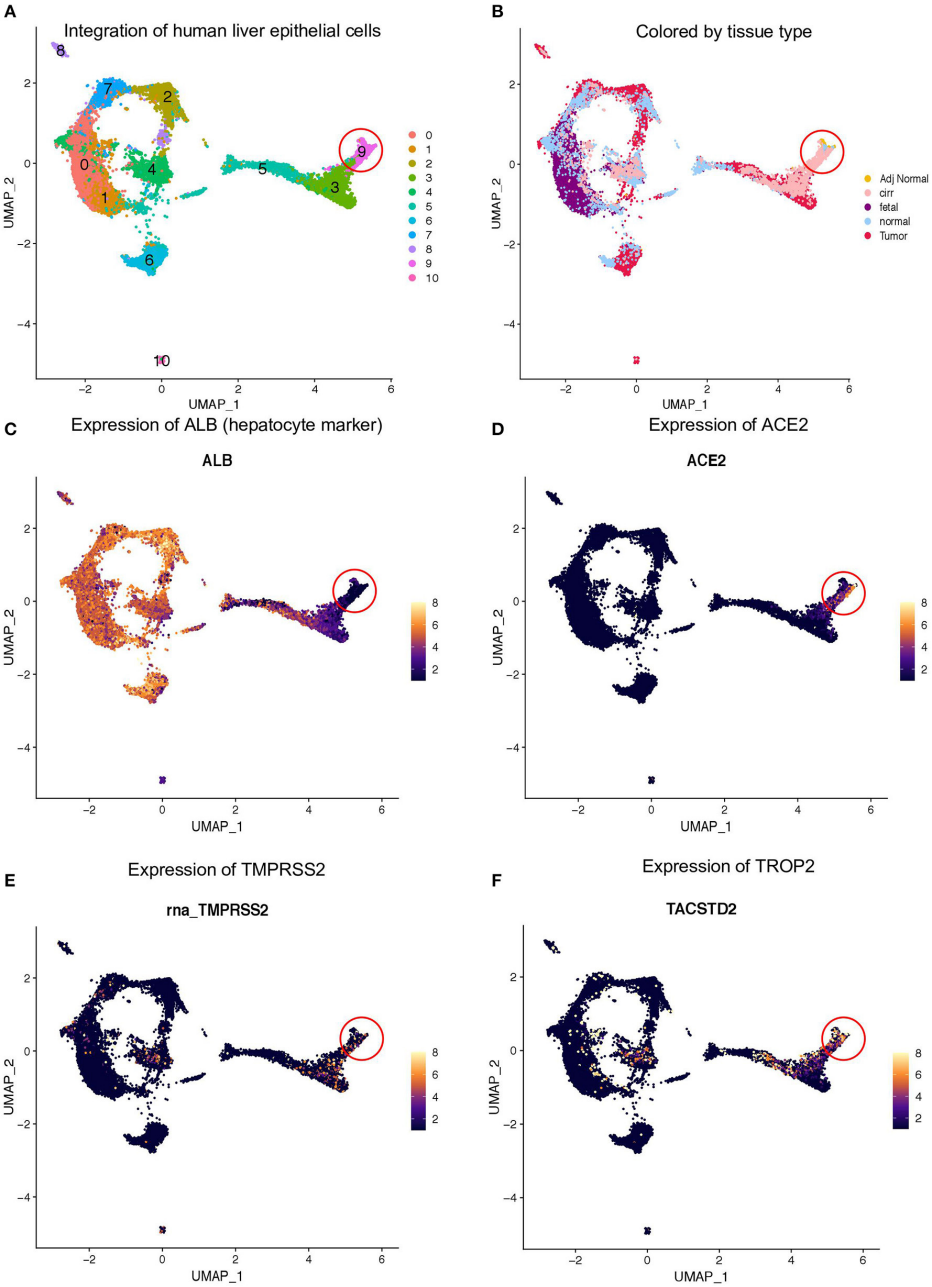
Expression of ACE2 and TMPRSS2 in liver epithelial cells. **(A)** Sub-clustering of epithelial cells from fetal, adult normal, cirrhotic, and HCC patients, which identified 11 clusters in the human liver. **(B)** Louvain clusters, colored by tissue types. Expression of **(C)** ALB, **(D)** ACE2, **(E)** TMPRSS2, and **(F)** TROP2.

**Figure 3:**
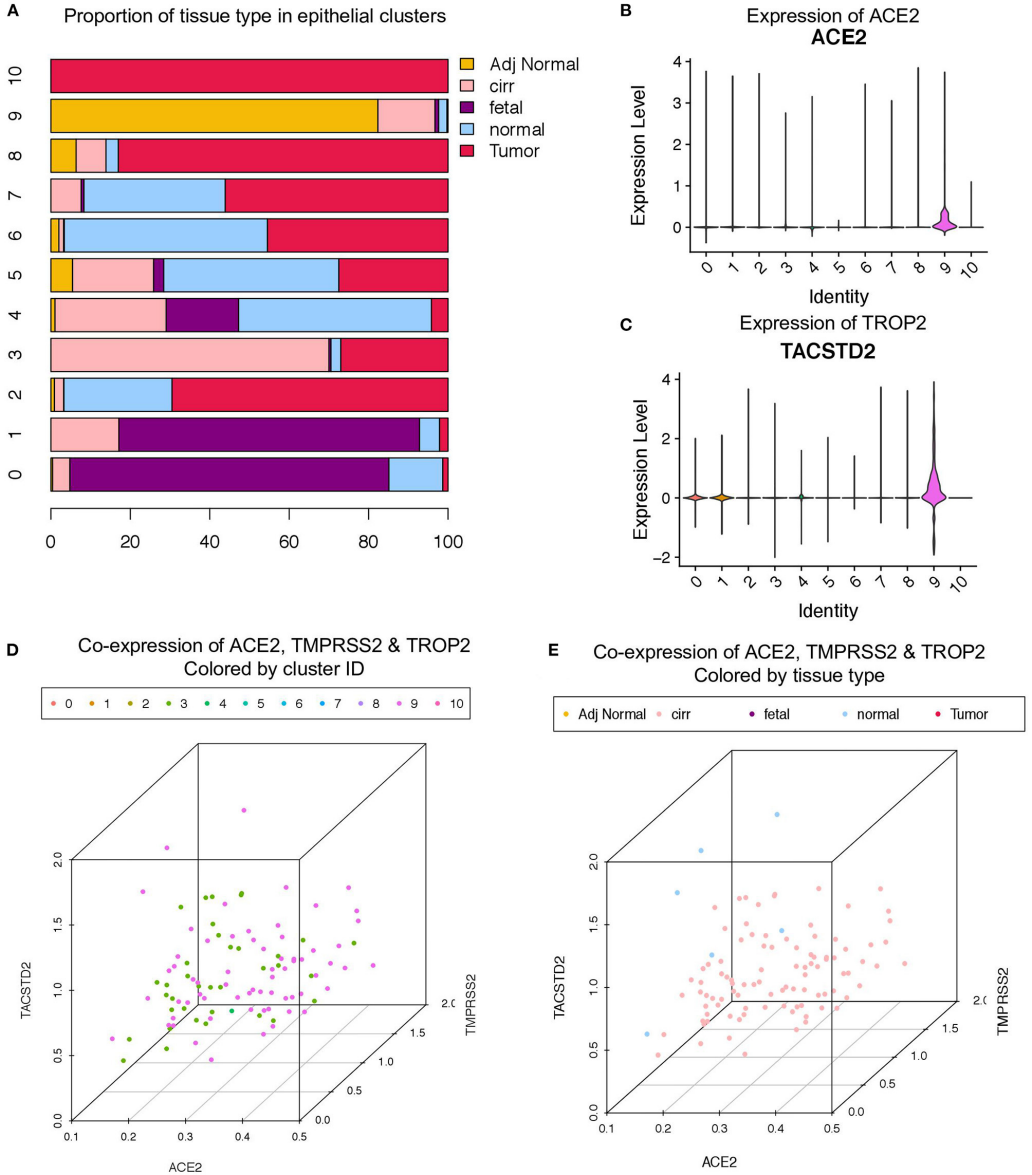
Co-expression of ACE2, TMPRSS2, and TROP2 in liver epithelial cells. **(A)** Bar plot depicting the proportion of different tissue types in liver epithelial clusters. Expression of **(B)** ACE2 and **(C)** TROP2 in Louvain clusters. Three-dimensional plots of ACE2, TMPRSS2, and TROP2 co-expression **(D)** colored by Louvain cluster ID and **(E)** colored by tissue type.

A recent scRNA-seq study has suggested heterogeneity in liver epithelial progenitors (21). Therefore, we further sub-clustered the epithelial cells (hepatocytes and progenitors) to understand the nature of ACE2 expressing liver progenitors (Figure 2A). Sub-clustering also showed the predominant presence of normal and cirrhotic liver cells in the progenitor cluster (cl. 9) (Figure 2B). Furthermore, we detected the absence of ALB in these cells, indicating a lack of differentiated cells in this cluster (Figure 2C). Interestingly, the lower abundance of these cells in the human liver is in concordance with the rare stem-like or progenitor population in epithelial tissues. We also detected the highest expression of ACE2, TMPRSS2, and TROP2 in cluster-9 (Figures 2D–F). Furthermore, we analyzed the proportion of cells from different tissue types in cluster-9 and observed the higher number of cells from adjacent normal and cirrhotic liver tissue (Figure 3A). Importantly, this is the only cell type that co-express ACE2 and TMPRSS2 in human liver single-cell atlas (Figures 3B,C). Finally, we analyzed the co-expression of ACE2, TMPRSS2, and TROP2 in human liver epithelial clusters and identified a higher proportion of ACE2^+^/TMPRSS2^+^/TROP2^+^ cells in cluster-9 and, more importantly, cirrhotic liver (Figures 3D,E).

We then analyzed the expression of hepatocyte, cholangiocyte, and bi-potent markers in these clusters (Supplementary Figure 1). The progenitor cluster specifically expressed EPCAM (progenitor marker) as well as KRT19 and cystic fibrosis transmembrane conductance regulator, which are known to be expressed in progenitors with a cholangiocyte fate bias (Supplementary Figure 1) (22). Importantly, we failed to detect the expression of hepatocyte fate bias genes, asialoglycoprotein receptor 1, and ALB in this cluster. As this progenitor cluster demonstrated bias for cholangiocyte fate, we further investigated the expression of the TROP2 gene. TROP2 expression is known to mark the fate of liver epithelial progenitors, where lower TROP2 expression is linked with hepatocyte fate and TROP2^high^ cells with cholangiocyte fate (21).

Recently, Aizarani et al. demonstrated the progenitor-like properties of TROP2^+^ cells, where TROP2^Int^ cells demonstrated the highest organoid-forming efficiency followed by TROP2^high^ cells, whereas TROP2^low^ cells failed to generate organoids (21). Therefore, we investigated whether any of the epithelial clusters co-expressed ACE2, TMPRSS2, and TROP2. Notably, we observed that only the EPCAM^+^ progenitor cluster expressed all three genes (Supplementary Figure 1E). We then subdivided this cluster into TROP2 low, intermediate, and high cells and investigated the expression of ACE2, TMPRSS2, and other cell fate markers. Remarkably, we observed that TROP2^high^ cells expressed the highest levels of ACE2 and TMPRSS2, followed by TROP2^Int^ and TROP2^low^ cells (Supplementary Figure 1F). Our analysis revealed that TROP2^Int^ (bi-potent) cells also express MUC6 and SOX9, whereas TROP2^high^ (cholangiocyte fate bias) cells express makers such as cystic fibrosis transmembrane conductance regulator, CXCL8, HES1, and KRT19. Our results suggest that SARS-CoV-2 can infect TROP2^high^ cells *via* ACE2 and TMPRSS2, thereby contributing to liver dysfunction by compromising the ability of the human liver to regenerate cholangiocytes.

### Enrichment of Angiotensin-Converting Enzyme 2 and Transmembrane Serine Protease 2 Co-expressing Cells in Cirrhotic Liver

**Figure 4:**
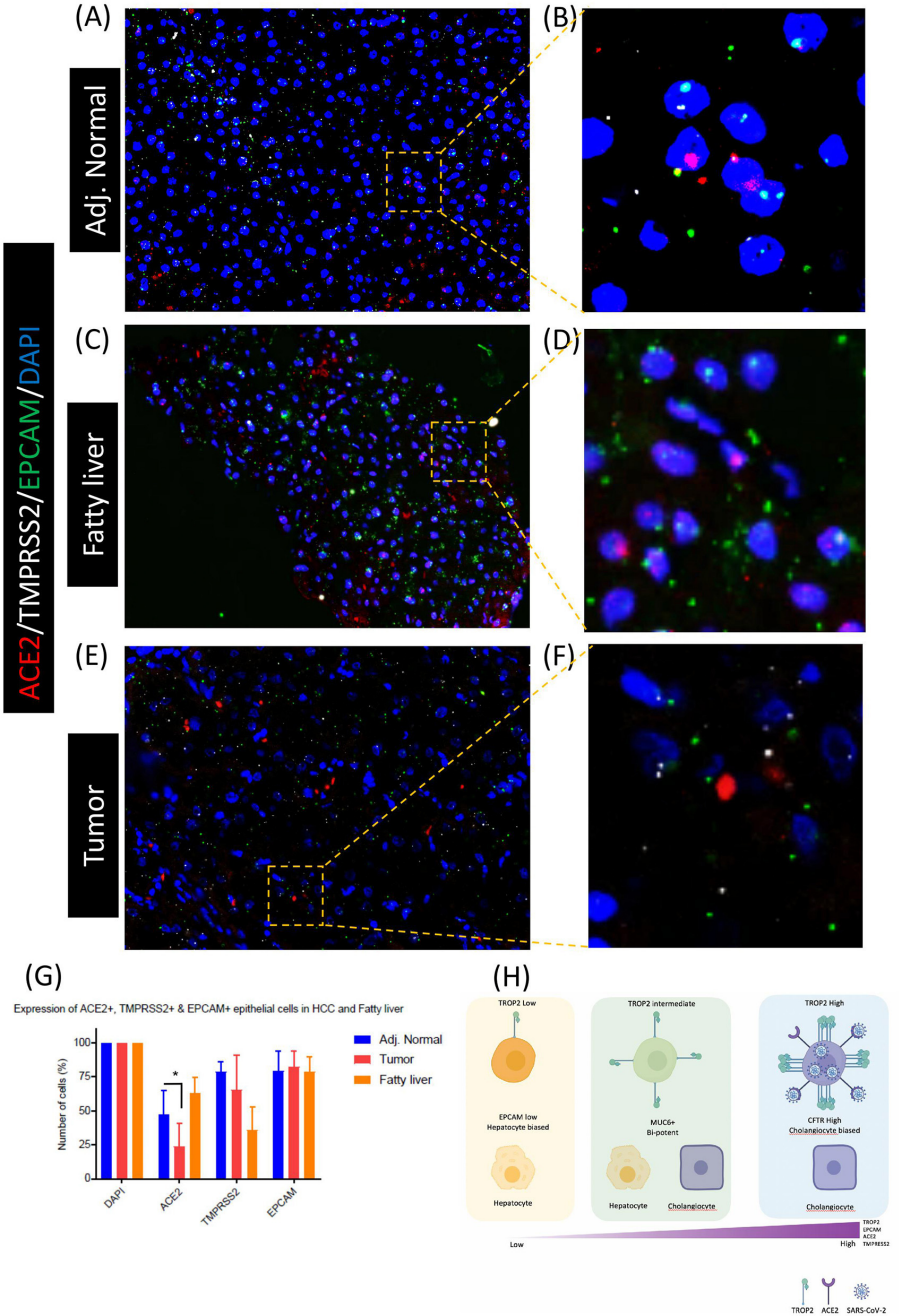
Co-enrichment of ACE2, TMPRSS2, and EPCAM in liver epithelial cells. RNA-FISH-based detection of ACE2, TMPRSS2, and EPCAM in **(A,B)** adjacent normal, **(C,D)** fatty liver, and **(E,F)** tumor tissues. **(G)** Quantification of RNA-FISH images. **(H)** Schematic of TROP2 expression level and cell fate choices in adult human liver progenitor cells. TROP2^high^ cells express genes exhibiting cholangiocyte fate bias. TROP2^high^ cells also express a higher level of ACE2 and TMPRESS2, making these cells more susceptible for SARS-CoV-2 infection, indicating implications in COVID-19-associated liver dysfunctions. *means statistical significance of *P* < 0.05.

As scRNA-seq analysis indicated a higher number of ACE2^+^/TMPRSS2^+^/TROP2^+^ co-expressing cells in cirrhotic liver, we used the RNA-FISH approach to validate these results in formalin-fixed paraffin-embedded (FFPE) tissues from the fatty (cirrhotic) liver, tumor, and adjacent normal sectors of hepatocellular carcinoma (Figures 4A–F). We probed the expression of ACE2, TMPRSS2, and Epcam in an RNA-FISH experiment and detected the higher number of Epcam^+^/ACE2^+^/TMPRSS2^+^ cells in fatty liver tissue when compared with adjacent normal and tumors (Figure 4G). Taken together, our results suggest that inflamed tissues such as the cirrhotic liver harbored a higher number of ACE2^+^/TMPRSS2^+^ epithelial progenitors when compared with normal and tumor tissues. These results indicate that patients with liver cirrhosis may have a higher probability of SARS-CoV-2 infection in the liver when compared with other individuals; this might worsen their regenerative abilities, leading to long COVID phenotypes.

## Discussion

In recent reports of the SARS-CoV-2 pandemic in the human population, the presence of viral messenger RNA in an infected patient's stool suggests a potential GI tract infection in COVID-19 patients. SARS-CoV-2 can reach the liver either through the general circulation once the patient has become viremic or through transmigration through the GI tract. We surveyed human liver scRNA-seq data to understand the expression pattern of the ACE2 and TMPRSS2 gene, which are essential for SARS-CoV-2 entry into human cells. Our analysis reveals that in the human liver, only EPCAM^+^ progenitors co-express genes for viral entry (ACE2) and S-protein priming (TMPRSS2). Further analyses revealed the specific expression of ACE2 and TMPRSS2 in TROP2^high^ cells. These results indicate that ACE2 and TMPRSS2 are specifically present in liver progenitors with a cholangiocyte fate bias, suggesting SARS-CoV-2 may be affecting cholangiocyte precursors, thereby potentially impeding the homeostasis of the cholangiocyte pool. Recent studies have reported the expression of ACE2 in cholangiocytes, however, they do not reflect on the heterogeneity of the ACE2^+^ population (23). The present study explored the heterogeneity of ACE2^+^ cells and systematically characterized ACE2 and TMPRSS2 co-expression as hallmarks of TROP2^+^ epithelial progenitors.

Our study reveals the potential of SARS-CoV-2 to infect TROP2^+^ progenitor-like cells in the cirrhotic liver. It is important to note that TROP2 is expressed in multiple epithelial progenitors (24–26). In the future, it will be important to survey other GI tract tissues at the single-cell level for the expression of ACE2 and TMPRSS2 and their associated transcriptomes. Given the GI tract infection and multi-organ failure in COVID-19, it is important to understand whether other progenitor-like cells are also susceptible to SARS-CoV-2 infection. Moreover, TROP2 expression has been associated with amplifying progenitor cells in the partial hepatectomy mouse model (24), indicating the important role of TROP2^+^ cells in liver regeneration. Taken together, our analysis suggests that cirrhotic human liver TROP2^+^ progenitors could be a prime target of SARS-CoV-2 (Figure 4H).

General hepatocyte cell damage from cytokine storms in ill patients with viremia from a respiratory viral infection is not uncommon. Such hepatocyte damage is usually transient, and the resulting liver regeneration usually restores liver function efficiently. In the case of COVID-19, however, the predilection of the SARS-CoV-2 virus for cholangiocyte precursor cells may significantly impair liver regeneration. Clinicians looking after patients with COVID-19 should be alerted to the possibility of progressive liver deterioration in patients with serious SARS-Cov-2 viremia. This study demonstrates the power of scRNA-seq to understand the pathobiology of COVID-19 and pave the way for similar studies to understand the effect of SARS-CoV-2 on different tissue and cell types.

## Experimental Methods

### Tissue Acquisition

Fresh tissue samples were obtained from Singapore General Hospital and National University with written consent and approval from the SingHealth Centralized Institutional Review Board (CIRB2012/669/B) to study liver cancer. The samples were delivered with MACS Tissue Storage Solution (Miltenyi, Cat#:130-100-008).

### Human Fetal Liver Samples

The donation of fetal liver tissues for research was approved by the Centralized Institutional Research Board of the Singapore Health Services in Singapore followed by proper international ethical guidelines and in accordance with a favorable ethical opinion from Singapore SingHealth and National Health Care Group Research Ethics Committees. Women gave written informed consent for the donation of fetal tissue to research nurses who were not directly involved in the research or the clinical treatments of women participating in the study, as per the Polkinghorne guidelines. This protocol was reviewed on an annual basis by the Centralized Institutional Research Board (IRB2013/837/D), including annual monitoring of any adverse events, for which there had been none. All fetal liver tissues were obtained from the second trimester (16 and 21 weeks estimated gestational age) elective pregnancy terminations carried out for sociopsychological reasons. All fetuses were considered structurally normal on ultrasound examination before termination and by gross morphological examination after termination. In total, 2 fetuses of 16 and 21 weeks estimated gestational age were used for this study.

### Tissue Processing

Tissues were transferred immediately and transferred to a sterile 10-mm^2^ tissue culture dish and cut into very small fragments. The dissociation buffer consisted of 0.43 mg/ml of collagenase IV (Thermofisher, Cat#: 17104019) and 0.172 mg/ul of DNAse1 (Worthington, Cat#: LS002147) dissolved in phosphate-buffered saline (PBS) (Thermofisher, Cat#: 20012-043). The tissue was digested in a dissociation buffer for 30–40 min depending on sample size at 37°C with constant shaking at 220 rpm while keeping the falcon tube in a slanted position. The solution was resuspended with a 10-ml pipette followed by an 18-g needle. The 1% bovine serum albumin (BSA) PBS solution was added to the digested tissue, and then, the solution was passed through a 70-um filter before centrifuging at 800 × *g* for 6 min at 4°C. Cells were treated with 5 ml of 1 × RBC lysis buffer (Biolegend, Cat#: 420301) on ice for 10–15 min. One percent BSA PBS solution was then added, and the cells were passed through a 40-um filter. Cells were dissolved in 1% BSA PBS solution before counting.

### RNA *in situ* Hybridization

FFPE slides of HCC and fatty liver samples were used in this experiment. Slides were stained using the RNAscope® Multiplex Fluorescent Reagent Kit v2 Assay Kit (Advanced Cell Diagnostics) following the manufacturer's protocol. The slides were baked in an oven at 60°C for 1 h. The slides were deparaffinized and dehydrated using fresh xylene (two washes for 5 min each) and fresh 100% ethanol (two washes for 2 min each). These deparaffinized slides were treated to RNAscope® Hydrogen Peroxide at room temperature for 10 min. The slides were placed in a slide holder with 200 ml of RNAscope® 1X Target Retrieval Reagent at 99°C for 15 min for target retrieval. The slides were allowed to cool down, and then, a hydrophobic barrier was drawn around the tissue with the ImmEdge™ hydrophobic barrier pen and was left to dry for around 5–10 min. Four to six drops of RNAscope® Protease Plus used for FFPE slides were added onto the slides and incubated at 40°C for 30 min. RNAscope probes from ACDbio were used in this experiment: ACE2 (Cat# 848151) in the C1 channel, TMPRSS2 (Cat# 470341) in the C2 channel, and EPCAM (Cat# 310281) in the C3 channel. Of the probe mix, 150–200 μl was added onto the slide, and probe hybridization was performed at 40°C for 2 h. The slides were then stored in 5 × SSC overnight, and amplification steps were performed the following day. For fluorescence, 1:500 dilution of Opal dyes (Perkin Elmer, Cat# NEL821001KT) was used, and the slides were mounted using a drop of ProLong Diamond Antifade Mountant (Thermofisher, Cat#: P36970). Imaging of slides was performed using Vectra® Polaris™ Automated Quantitative Pathology Imaging System.

Image quantification was done using ImageJ and Cell Profiler software. Using the ImageJ software, the images were split into single-channel grayscale images. These grayscale images were added onto Cell Profiler software and analyzed using a published pipeline (27).

### Data Processing Using Cell Ranger Software

Sequenced fastq files are aligned, filtered, barcoded and UMI counted using Cell Ranger Chromium Single Cell RNA-seq version 2.0.2, by 10 × Genomics with Cell Ranger, GRCh38 database (version 1.2.0) as the human genome reference. All 62 sectors are aggregated using *cellranger aggr* by normalizing all runs to the same sequencing depth.

### Clustering and Downstream Analysis

Downstream analysis was performed using Scanpy, a scalable Python-based package (version 1.4) designed for single-cell gene expression datasets. Scanpy implements numerous functions from preprocessing to visualization, clustering, differential gene expression, and trajectory inference analysis on Jupyter Notebooks. Parameters used in each function are manually curated to portray the best clustering of cells. In preprocessing, cells are filtered based on the criteria of expressing a minimum of 200 genes and a gene that is expressed by a minimum of 30 cells. Dying cells with a mitochondrial percentage of more than 5% are excluded. Cell count was normalized using *scanpy.api.pp.normalize_per_cell* with a scaling factor of 10,000, whereas gene expression was scaled to unit variance and mean value of 0 using *scanpy.api.pp.scale*. Dimension reduction starts with PCA using *scanpy.api.tl.pca*; the number of PCs used in each clustering exercise varies depending on the importance of embeddings to be included. In the interest of crisp clustering, we first calculated the neighborhood graph (*scanpy.api.pp.neighbors*) of cells. Best matched k-Nearest Neighbor is automatically weighted by the algorithm to compute the best UMAP topology (*scanpy.api.tl.umap*, minimum distance between 0.3 and 0.5) which is consistently used throughout this paper. Louvain method (*scanpy.api.tl.louvain*) is then used to detect a community of similar cells. By default, Louvain's resolution parameter is set to the maximum value of 1.0; this, in theory, finds more and smaller clusters. In our experiments, the value is set between 0.6 and 1. Genes are then ranked using *scanpy.api.tl.rank_genes_groups* (Benjamini–Hochberg, *t*-test overestimated variance with adjusted *p*-value). Cell types were manually and iteratively assigned based on overlaps of literature curated and statistically ranked genes. To leverage the heterogeneity of this dataset, we used partition-based graph abstraction (*scanpy.api.tl.paga*) to reconstruct lineage between cell types. This lineage trajectory provides a continuous cell type transition from the assigned discrete cell types. The thickness of the edges represents connectivity scores, an entropy-based measure provided by partition-based graph abstraction indicating the relatedness between clusters; spurious connections are discarded while tuning thresholds.

### Statistical Analysis

The statistical analysis for the image quantification was performed in Prism 7 (GraphPad). Data are expressed as mean ± SEM.

## Data Availability Statement

The datasets presented in this study can be found in online repositories. The names of the repository/repositories and accession number(s) can be found at: https://www.ncbi.nlm.nih.gov/, GSE156337.

## Ethics Statement

The studies involving human participants were reviewed and approved on an annual basis by the Centralized Institutional Research Board (IRB2013/837/D), including annual monitoring of any adverse events, for which there had been none. Written informed consent to participate in this study was provided by the participants' legal guardian/next of kin.

## Author Contributions

AS, PC, FG, and RD: conceptualization. AS and JS: methodology, formal analysis, and data curation. AS, JS, and AM: investigation. AS: writing—original draft and supervision. PC, FG, and RD: writing—review and editing. PC, FG, AS, and RD: funding acquisition. JC, TL, BG, PC, FG, and RD: resources. All authors contributed to the article and approved the submitted version.

## Conflict of Interest

The authors declare that the research was conducted in the absence of any commercial or financial relationships that could be construed as a potential conflict of interest.
