# Supplementary material for: Single-Cell RNA-seq Reveals Angiotensin-Converting Enzyme 2 and Transmembrane Serine Protease 2 Expression in TROP2+ Liver Progenitor Cells: Implications in Coronavirus Disease 2019-Associated Liver Dysfunction
Source: Front Med (Lausanne). 2021 Apr 22;8:603374. doi: 10.3389/fmed.2021.603374 (PMC8100026; doi:10.3389/fmed.2021.603374)

Supplementary Figure-1

Figure-2

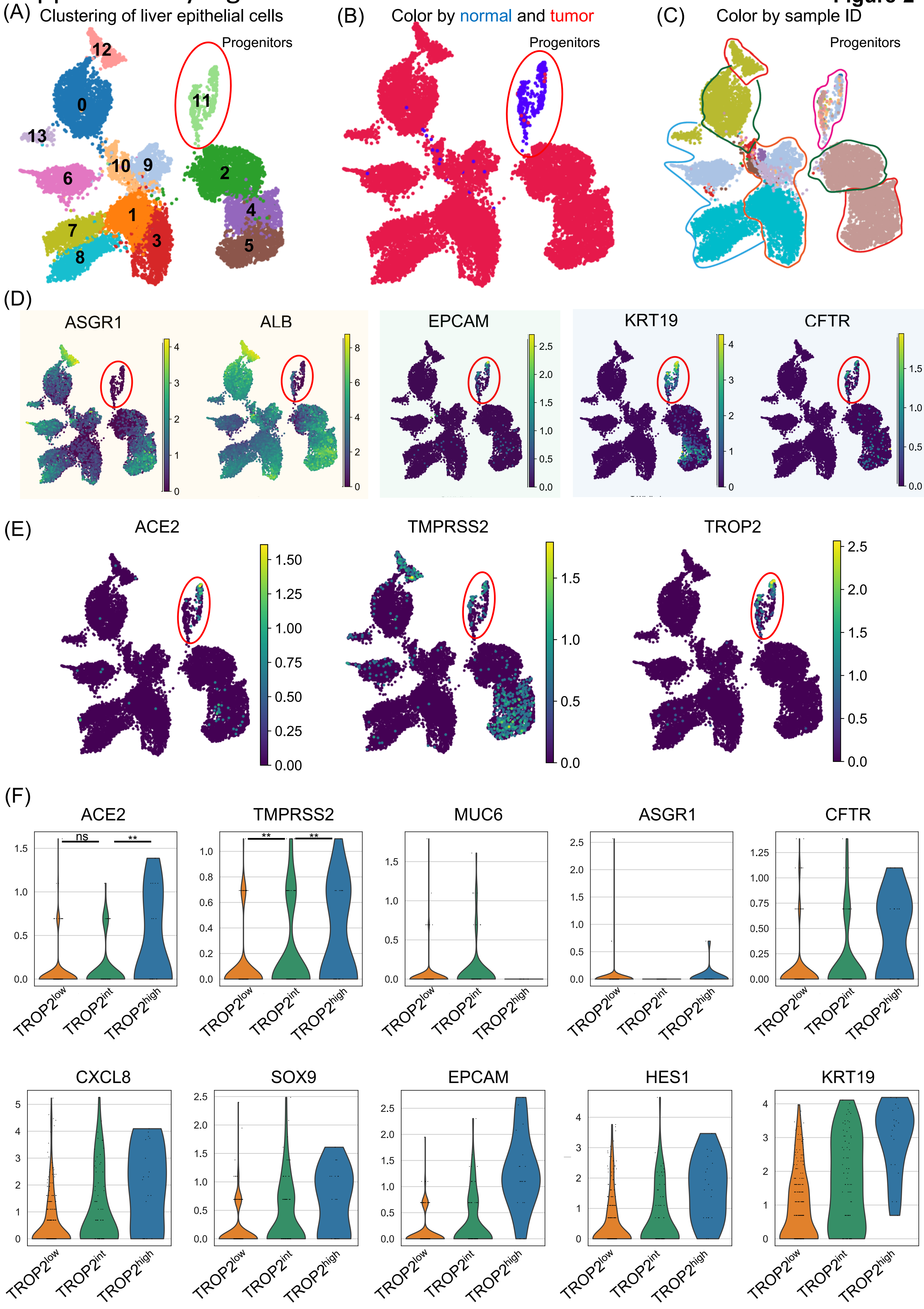

Supplement: Supplementary file 1 [file Image_1.pdf]
