# Supplementary material for: Single-Cell RNA-seq Reveals Angiotensin-Converting Enzyme 2 and Transmembrane Serine Protease 2 Expression in TROP2+ Liver Progenitor Cells: Implications in Coronavirus Disease 2019-Associated Liver Dysfunction
Source: Front Med (Lausanne). 2021 Apr 22;8:603374. doi: 10.3389/fmed.2021.603374 (PMC8100026; doi:10.3389/fmed.2021.603374)
Supplement: Supplementary file 2 [file Data_Sheet_1.PDF]

Supplementary Figure-1 Expression of ACE2 and TMPRSS2 in TROP2^{+} liver progenitors

(A) Louvain clustering of ~10,000 sc-RNA-seq libraries from epithelial cells identifies 12 sub-clusters in human liver. (B) Louvain clusters colored by normal (blue) and tumor (red) cell types. Note the predominate normal cells in progenitor cluster. (C) Louvain clusters colored by sample ID note the representation of multiple sample IDs in progenitor cluster. (D) Expression of hepatocyte fate biased (ASGR1 and ALB), bi-potent (EPCAM) and cholangiocyte fate biased (KRT19 and CFTR) genes in progenitor cluster (circled red). (E) Expression of ACE2, TMPRSS2 and TROP2 in progenitor cluster (circled red). (F) Expression of ACE2 and TMPRSS2 in TROP2^{low} (orange), TROP2^{int} (green) and TROP2^{high} (blue) cells (note the highest expression of ACE2 and TMPRSS2 in TROP2^{high} cells as well as expression of other cell fate markers). (The progenitor cluster was binned into TROP2^{low} (orange), TROP2^{int} (green) and TROP2^{high} (blue) cells).
